# The many forms of a pleomorphic bacterial pathogen—the developmental network of *Legionella pneumophila*

**DOI:** 10.3389/fmicb.2014.00670

**Published:** 2014-12-22

**Authors:** Peter Robertson, Hany Abdelhady, Rafael A. Garduño

**Affiliations:** ^1^Department of Microbiology and Immunology, Dalhousie UniversityHalifax, NS, Canada; ^2^Division of Infectious Diseases, Department of Medicine, Dalhousie UniversityHalifax, NS, Canada

**Keywords:** differentiation, developmental forms, intracellular infection, disease transmission, pathogen detection

## Abstract

*Legionella pneumophila* is a natural intracellular bacterial parasite of free-living freshwater protozoa and an accidental human pathogen that causes Legionnaires' disease. *L. pneumophila* differentiates, and does it in style. Recent experimental data on *L. pneumophila*'s differentiation point at the existence of a complex network that involves many developmental forms. We intend readers to: (i) understand the biological relevance of *L. pneumophila*'s forms found in freshwater and their potential to transmit Legionnaires' disease, and (ii) learn that the common depiction of *L. pneumophila*'s differentiation as a biphasic developmental cycle that alternates between a replicative and a transmissive form is but an oversimplification of the actual process. Our specific objectives are to provide updates on the molecular factors that regulate *L. pneumophila*'s differentiation (Section The Differentiation Process and Its Regulation), and describe the developmental network of *L. pneumophila* (Section Dissecting *Lp*'s Developmental Network), which for clarity's sake we have dissected into five separate developmental cycles. Finally, since each developmental form seems to contribute differently to the human pathogenic process and the transmission of Legionnaires' disease, readers are presented with a challenge to develop novel methods to detect the various *L. pneumophila* forms present in water (Section Practical Implications), as a means to improve our assessment of risk and more effectively prevent legionellosis outbreaks.

## Background

### *L. pneumophila* is a facultative intracellular pathogen that differentiates into numerous forms within a developmental network

*Legionella pneumophila* (*Lp*) is an intracellular bacterial pathogen predicted to have co-evolved with freshwater protozoa (Barker and Brown, 1994; Weissenberger et al., 2007; Garduño, 2008) to optimize the acquisition of intracellular nutrients (Price et al., 2014). The fact that *Lp* can grow outside host cells, either in nutrient-rich media *in vitro*, or within microbial communities (reviewed by Declerck, 2010), technically defines it as a facultative intracellular pathogen. However, in nature, *Lp* behaves more as an obligate intracellular pathogen and less as a facultative one. That is, in relation to growth inside natural hosts, extracellular replication represents but a minor contribution (Temmerman et al., 2006) to the maintenance of *Lp* populations in freshwater, or to the increase of bulk *Lp* levels (Murga et al., 2001; Kuiper et al., 2004; Declerck et al., 2007, 2009; Fields, 2008). Consequently, intracellular growth is considered a fundamental process in the life cycle of *Lp* in general, and *Lp* differentiation in particular (Garduño, 2008). Amoebae are the preferred *Lp* hosts in the natural environment. Fifteen amoebal species have been reported to support the intracellular growth and differentiation of *Lp* (Hägele et al., 2000, and reviewed by Fields, 1996, 2008).

We have previously discussed the intracellular differentiation of *Lp* (Garduño, 2008), and established that *Lp* has a single developmental program integrated into its life cycle (Garduño et al., 2008), with 14 *Lp* developmental forms reported to date (Rowbotham, 1980; Gress et al., 1980; Faulkner and Garduño, 2002; Greub and Raoult, 2003; Sauer et al., 2005a; Faulkner et al., 2008; Al-Bana et al., 2014) (Table 1). Given the complexity of *Lp*'s ecology and the many developmental forms involved, we also proposed the existence of a developmental network (Garduño et al., 2008). This developmental network includes the “accidental” hosts that support the intracellular growth of *Lp* in the context of laboratory investigations, or in the context of human Legionnaires' disease. In this review we will discuss the developmental network of *Lp* and provide as many details as possible, about the many developmental forms that *Lp* produces.

**Table 1:** **The *Lp* developmental forms that have been identified and reported to date**.

**Name used (abbreviation)**	**Main characteristics**	**Primary references**
Exponential phase form (EPF)	Produced extracellularly, non-infectious to host cells, sensitive to stress, replicates actively	Byrne and Swanson, 1998
Stationary phase form (SPF)	Produced extracellularly, infectious to host cells, resistant to stress	Byrne and Swanson, 1998
Filamentous form (FF)	Produced extra- and intra-cellularly, infectious to host cells, forms dense biofilms	Rodgers et al., 1978; Piao et al., 2006
Mature infectious form (MIF)	Produced intracellularly, infectious to host cells, resistant to stress	Garduño et al., 2002
Immature intracellular form (IIF)	Produced in cultured macrophages, morphologically undifferentiated, less infectious and less resistant to stress than MIFs, elongated	Abdelhady and Garduño, 2013
Replicative phase form (RPF)	Produced intracellularly, replicates actively	Faulkner and Garduño, 2002
MIF-EPF intermediate	Produced extracellularly upon germination of mature infectious forms in BYE, shows intraperiplasmic vesicles	Faulkner and Garduño, 2002
MIF-RPF intermediate	Produced intracellularly in response to the presence of amino acids, a precursor to the initiation of replication in the LCV^a^	Sauer et al., 2005a
RPF-MIF intermediates	Produced intracellularly in the late stages of the infection cycle, display unique envelope profiles. Might be similar to IIFs	Faulkner and Garduño, 2002
VBNCC^a^ derived from a SPF	Produced extracellularly in response to sustained stress, resuscitates in the presence of amoeba	Steinert et al., 1997; Al-Bana et al., 2014
VBNCC derived from a MIF	Produced extracellularly in response to stress, shows an intact cell ultrastructure, does not resuscitate in amoeba	Al-Bana et al., 2014
VBNCC derived from an EPF	Apparently more fragile than the other VBNCCs mentioned above	Ohno et al., 2003
Pelleted MIFs	Produced by ciliates and amoeba, show unique developmental traits	Berk et al., 1998, 2008
Pelleted VBNCCs	Produced by ciliates, may show unique developmental traits	Al-Bana et al., 2014

*^a^Abbreviations used: LCV, Legionella-containing vacuole; VBNCC, viable but non-culturable cell*.

### An overview of the developmental cycles and the developmental network of Lp

**Figure 1:**
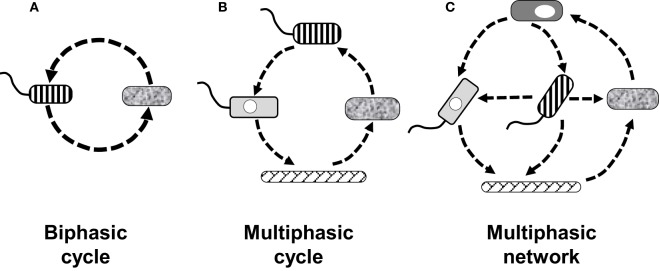
**Diagrammatic examples of how the number of forms and their differentiation links define different types of developmental cycles, or a developmental network**. **(A)** Biphasic cycle in which two forms simply alternate into each other. **(B)** Multiphasic cycle showing four forms giving rise to each other in a sequential (linear) manner. **(C)** Multiphasic network of five forms in which the differentiation links are not linear. The differentiation network of *L. pneumophila* includes 14 recognized forms, to date, which are developmentally linked in a non-linear fashion, making the network highly complex.

Key to the establishment of a developmental cycle is the demonstration that the various forms present in it can differentiate into each other, closing a circular process. In the simplest cycle (a biphasic one) one originating form gives rise to another, which in turn differentiates back into the originating one (Figure 1). In the case of a multiphasic cycle, more than two forms would sequentially differentiate into each other. When the differentiation links are not sequentially circular a developmental network is then established (Figure 1).

#### Extracellular vs. intracellular Lp's developmental cycles

In Section *L. pneumophila* is a Facultative Intracellular Pathogen that Differentiates into Numerous Forms within a Developmental Network, we already defined *Lp* as a facultative intracellular pathogen. Therefore, when discussing the *Lp* differentiation process we need to consider, distinguish and compare the differentiation steps that happen extracellularly, in relation to those linked to intracellular growth or residence. The extracellular development of *Lp* follows a biphasic cycle involving replicative exponential phase forms (EPFs) and transmissive stationary phase forms (SPFs) (reviewed by Molofsky and Swanson, 2004), with differentiation links to viable but non-culturable cells (VBNCCs) and filamentous forms (FFs) (Table 1).

Intracellularly, *Lp*'s developmental cycles are multiphasic. Ever since we first described *Lp*'s multiphasic cycle in HeLa cells (Faulkner and Garduño, 2002), we have hypothesized that *Lp* follows many intracellular multiphasic developmental cycles, one per host cell type (Garduño, 2008). This hypothesis stemmed from morphological observations suggesting that *Lp* reaches different developmental endpoints in different host cells (Garduño et al., 2002) and has been now confirmed. That is, we showed that the *Lp* progeny produced in *Acanthamoeba castellanii* is morphologically differentiated and infectious to cells in culture, whereas the progeny produced in human macrophages derived from the U937 or THP-1 cell lines was only partially differentiated (morphologically) and showed infectivity defects (Abdelhady and Garduño, 2013). In addition, as *Lp* interacts with ciliated protozoa of the genus *Tetrahymena* at a temperature of 30°C or lower, it does not replicate (Berk et al., 2008), but still differentiates intracellularly (Faulkner et al., 2008), establishing yet a different developmental cycle with a unique endpoint (Section The Cycle of Packaged *Lp* Forms below).

#### The developmental network of Lp and Why it is necessary to dissect it

In *Lp*'s developmental network, forms within one given developmental cycle, also differentiate into developmental forms that typically belong to another cycle. We refer readers to Figure 4.4 from the Garduño et al. (2008) review, to sample the complexity of the developmental network of *Lp*, as we understood it then. In this review, we will refrain from trying to represent the entire *Lp*'s developmental network, as we currently understand it, in one single figure as it would be too difficult to fit. Instead, in Section Dissecting *Lp*'s Developmental Network we present five cycles that when pieced together should provide a fair representation of the entire developmental network of *Lp*.

## The differentiation process and its regulation

### Key molecular players in the differentiation network of Lp—an update

**Figure 2:**
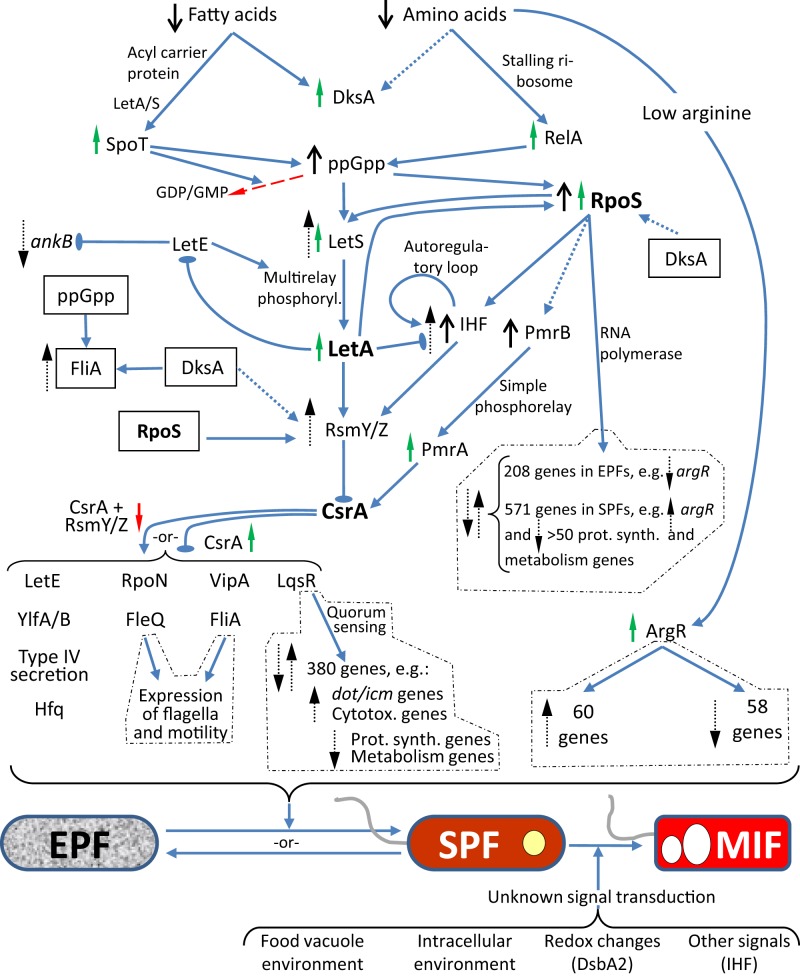
**Diagram showing the regulatory pathways of *Lp* differentiation as described in Section The Differentiation Process and Its Regulation of the text**. Known confirmed interactions are depicted by blue solid arrows, where pointy arrows indicate positive/inducing effects and oval-headed arrows negative/repressing effects. Dotted blue lines indicate experimentally unproven or indirect links. Master regulators are shown in boldface. The boxed factors were duplicated to be placed in a convenient position to show additional links. RpoS, FleQ, and FliA (regulators of class II, III and class IV flagellar genes, respectively), LqsR (through quorum sensing) and ArgR have their own transcriptional regulatory networks (dotted line boxes). The *Lp* forms EPF, SPF, and MIF are as per Table 1. The top pathways pertain to the EPF-to-SPF differentiation in a low nutrient environment, but the interactions can be reversed to show the SPF-to-EPF differentiation in a nutrient-rich environment. Black arrows indicate high or low factor levels. Red and green arrows indicate decreased or increased activity of the corresponding factor, respectively. Black dotted line arrows indicate upregulation or downregulation of transcription. Besides being a ppGpp synthase, SpoT is also a ppGpp hydrolase, and this activity is depicted by the red dashed arrow.

Differentiation of *Lp* may be implicitly viewed as an adaptation to radically different intracellular and extracellular environments, thus requiring the timely coordination of gene expression to achieve useful phenotypical traits. Not surprisingly, the key molecular players in *Lp* differentiation are regulators that directly or indirectly control the expression of virulence and fitness factors at the transcriptional and (or) post-transcriptional levels. These key molecular players are part of the regulatory pathways shown in Figure 2, but it should be acknowledged that these pathways are still not fully elucidated.

#### ppGpp, RelA, and SpoT

The alarmone guanosine 3′-diphosphate-5′-diphosphate, or ppGpp, is a recognized trigger of the stringent response of bacteria. Although best studied in *E. coli* (Magnusson et al., 2005), ppGpp is key for the differentiation of *Lp* from EPFs to SPFs (Hammer and Swanson, 1999). Produced in response to low nutrient levels, ppGpp is capable of (among a plethora of actions) binding RNA polymerase and altering the polymerase's preference for alternate sigma factors and promoters, and consequently, changing gene expression profiles (Artsimovitch et al., 2004; Magnusson et al., 2005; Potrykus and Cashel, 2008; Dalebroux and Swanson, 2012; Ross et al., 2013). RelA and SpoT are the two enzymes known to synthesize ppGpp in *Lp*, in response to distinct nutritional conditions (Zusman et al., 2002; Dalebroux et al., 2009). In addition, SpoT is a ppGpp hydrolase, responsible for reducing ppGpp levels in *Lp* (Dalebroux et al., 2009). Whereas RelA is a ribosome-associated enzyme that gets activated as a consequence of the ribosomal engagement of uncharged tRNAs (Haseltine et al., 1972; Wendrich et al., 2002), SpoT seems to be activated by a reduction in the rate of fatty acids biosynthesis and(or) increased concentrations of short chain fatty acids (Dalebroux et al., 2009; Edwards et al., 2009). In both *E. coli* and *Lp* the stringent response typically results in upregulation of genes involved in stress resistance and virulence, and a downregulation of genes involved in growth and proliferation. The difference between the stringent responses of these two organisms thus relies on context rather than function. Whereas *E. coli* uses the stringent response primarily to overcome adverse conditions, *Lp* has integrated this response into survival and differentiation. As well, high levels of ppGpp are known to increase the stability and activity of alternative sigma factors including RpoS, one of the major regulators of the stationary phase in *Lp* (reviewed by Dalebroux and Swanson, 2012).

In *Lp*, synthesis of ppGpp seems to be as important as its hydrolysis, as demonstrated by both the fact that *spoT* mutants cannot be obtained in the presence of a functional RelA, and the inability of double *spoT relA* mutants complemented with either a fully functional RelA, or a defective SpoT, to resume intracellular growth, or growth in a nutrient-rich medium (Dalebroux et al., 2009). In both *Lp* and *E. coli* the ability to monitor fatty acid biosynthesis is through an interaction between SpoT and acyl carrier protein, and in *Lp* a functional LetA/S system (see below) is also required (Dalebroux et al., 2009; Edwards et al., 2010). Comparisons between the *relA spoT* double mutant and a *relA* mutant also demonstrate that SpoT plays a role in differentiation, likely as a consequence of the very low levels of ppGpp in the *relA spoT* double mutant (Dalebroux et al., 2009).

#### DksA

Although ppGpp is a major key inducer of differentiation, other factors act in concert with it to modify, enhance and (or) control the process. DksA is a ribosome-binding protein that acts together with ppGpp to modify the initiation of transcription. Although *dksA* deletion mutants still amass high levels of ppGpp, they are deficient at inducing virulence, cytotoxicity to macrophages, sodium sensitivity and motility (i.e., transmissive traits) in SPFs (Dalebroux et al., 2010). Furthermore, the *dksA* deletion mutant is unable to differentiate in response to propionic acid (a chemical that disturbs fatty acid metabolism). That is, when exposed to propionic acid *dksA* mutants were deficient in motility and escape from phagosomes, suggesting that full differentiation in response to fatty acid perturbations requires DksA. The aforementioned deficiencies are attributed to the absence of DksA-mediated modification of gene expression, as DksA is known to enhance the expression of *fliA*. *flaA*. *rsmZ*, and other regulatory components (Dalebroux et al., 2010). For instance, inducible expression of *dksA* from a plasmid restored 20–35% motility in a ppGpp-negative, non-motile *Lp* strain, enhancing transcription of *fliA* and *flaA*. DksA also appears to be necessary (at least partially) for survival of *Lp* in stationary phase, as *dksA* mutants grow normally in exponential phase but lose viability in stationary phase relative to the parent *Lp* strain (Dalebroux et al., 2010). In summary, DksA participates in (and complements) ppGpp-mediated processes. Further work on DksA is warranted, as its impact on *Lp* differentiation is still poorly understood.

#### RpoS

The alternative RNA polymerase sigma factor RpoS is considered a master regulator of *Lp* differentiation from replicative forms (EPFs or RPFs) into transmissive forms (SPFs or MIFs), and has been studied in great extent. In our previous review (Garduño et al., 2008), we presented the major known properties of the *Lp* RpoS, and highlighted the fact that the regulation of *rpoS* expression and RpoS activity, constitutes a complex process different from that described in *E. coli*. Here we will focus on recent (after 2008) data.

The ppGpp-dependant induction of RpoS (see above) is also dependent on DksA in *E. coli* and *Salmonella enterica*, as the deletion of *dksA* results in significantly lower levels of RpoS, even when ppGpp is produced (Brown et al., 2002; Paul et al., 2004). However, it is not known yet whether expression of the *Lp* RpoS is also dependent on DksA. There can be little doubt of the centrality of RpoS to *Lp* differentiation as recent microarray data has confirmed that RpoS significantly alters gene expression, affecting 208 genes in exponential phase, and 571 genes in stationary phase (Hovel-Miner et al., 2009). The expression of genes encoding for secretion substrates of the type IV virulence-related secretion system, Dot/Icm, is modified both positively and negatively in both growth phases, suggesting that RpoS coordinates a shift in the effectors that are released at different stages of the infection cycle (Hovel-Miner et al., 2009). *Lp* RpoS also caused the downregulation in SPFs of over 50 genes related to translation and metabolism. Expression of the small RNAs *rsmY* and *rsmZ* is also enhanced by RpoS, leading to an increase in transmissive traits via the sequestration of CsrA (see below) (Rasis and Segal, 2009). Finally, *rpoS* mutants are unable to differentiate into MIFs in HeLa cells, and either get digested in the intracellular environment of *T. tropicalis* (Faulkner et al., 2008) or do not grow in amoebae (Hales and Shuman, 1999; Abu-Zant et al., 2006).

Although the *Lp* RpoS is clearly important for *Lp* differentiation in stationary phase, we previously discussed that it also plays a functional role in the exponential growth phase. In fact, some of the genes upregulated by RpoS in stationary phase are actually downregulated by RpoS during the exponential growth phase, as confirmed for the metabolic gene *argR* which enhances intracellular growth in amoebae, but not in macrophages (Hovel-Miner et al., 2009). *Lp*'s ArgR is a transcriptional regulator with an unusually large and complex regulon (Hovel-Miner et al., 2010). In the stationary growth phase, ArgR positively affects the expression of 60 genes, and negatively affects the expression of 58 genes, in response to exogenous low arginine concentrations (Hovel-Miner et al., 2010).

#### Two-component regulatory systems (LetA/S, PmrA/B, and, LqsR/S)

The *Lp* LetA/S system is a two-component regulatory system (2CRS) whose activity is required for full expression of transmissive phenotypes in SPFs. LetA/S is part of the signal transduction pathway that connects nutritional gaging to differentiation responses, and occupies a crossroads position between ppGpp, RpoS, and CsrA (see below and Figure 2). Not surprisingly, the LetA/S system was determined to be involved in the regulation of *rpoS*, several *icm* genes, *flaA*. *plaC*, and other genes involved in lipid metabolism, *ralF* and *hfq* (Gal-Mor and Segal, 2003; Lynch et al., 2003; McNealy et al., 2005; Broich et al., 2006). Unlike most 2CRSs, the *Lp* LetA/S system acts as a rheostat (rather than an ON/OFF switch) by virtue of including multiple phosphorylation steps in the phosphorelay pathway, thus making it comparable to the BvgA/BvgS 2CRS of *Bordetella pertussis* (Edwards et al., 2010). High levels of ppGpp activate LetA/S, but the transcription of *letS* is reduced in an *rpoS* mutant, confirming that multiple factors (including LetE) are actually combined to increase the expression and activity of the LetA/S system (Hovel-Miner et al., 2009). The effects of the LetA/S system on induction of transmissive phenotypes are indirect, as LetA/S acts by repressing CsrA (Sahr et al., 2009) by virtue of inducing the expression of the small RNAs *rsmY* and *rsmZ* (Rasis and Segal, 2009), which in turn bind to CsrA to antagonize its activity (see Section CsrA below).

While the precise link between the LetA/S system and LetE remains unsolved, recent information indicates that LetA appears to repress expression of *letE*, as levels of LetE increase three-fold in a *letA* mutant (Sahr et al., 2009). LetE is also known to repress *ankB*, giving LetE both positive and negative roles in regulation (Al-Khodor et al., 2008). As indicated above for RpoS, *Lp letA* mutants do not differentiate into MIFs and are digested in *Tetrahymena*, but are able to establish a *Legionella*-containing vacuole and grow well in HeLa cells (Faulkner et al., 2008). In addition, it is known that a *letA* mutant grows well in human macrophages, but not in *A. castellanii* (Gal-Mor and Segal, 2003).

The PmrA/B 2CRS is used in *Lp* to control Dot/Icm secretion and the stress response (Zusman et al., 2007). It has been suggested that the PmrA/B system may have a more global effect on transcription in *Lp*, as microarray data shows that mutations in *pmrA/B* cause the differential expression of 279 genes, including type IV and type II secretion effectors, stress response genes, metabolic genes, and others (Al-Khodor et al., 2009). The PmrA/B system's link to the differentiation regulatory network comes from the facts that *pmrA/B* mutants exhibited significantly lower levels of *csrA* in both the exponential and stationary growth phases (Al-Khodor et al., 2009), and that *pmrA/B* is regulated by RpoS (Hovel-Miner et al., 2009). Unfortunately, no mechanism for this role has been elucidated at this time.

Finally, the Lqs 2CRS responsible for quorum sensing in *Lp* is also involved in regulating differentiation. Three gene products are key for the function of this system, LqsA (encoding the autoinducer synthase), LqsS (encoding the sensor kinase) and LqsR (encoding the response regulator) (Tiaden et al., 2008). Expression of LqsR is growth phase-dependent and controlled by the combined action of RpoS and LetA (Tiaden et al., 2007), likely through RsmY/Z and CsrA (Section CsrA and Figure 2). The Lqs system, in turn, controls the expression of more than 380 genes; of relevance being the upregulation of virulence traits in SPFs (Tiaden et al., 2008). The most recent piece of information regarding the Lqs system is that LqsT, a second sensor kinase besides LqsS, is capable of phosphorylating (and activating) LqsR (Schell et al., 2014). Besides the autoinducer, the signals to which the two sensor kinases respond are not yet elucidated, but their convergence upon a single response regulator strongly suggests an unusual flexibility with enhanced signaling options.

#### CsrA

The carbon storage regulator CsrA is an RNA-binding protein that recognizes a binding site near the 5′ end of target transcripts (Baker et al., 2002) and blocks their translation. In EPFs, CsrA blocks the translation of transcripts encoding transmissive traits and stationary phase-associated factors, including *rpoN*. *fliA*. *letE*. *ylfA*/*B*, and *vipA* (Forsbach-Birk et al., 2004; Rasis and Segal, 2009). During the stationary growth phase, the LetA/S system is activated by the combined action of ppGpp and other factors (see above), and induces production of the non-coding RNAs RsmY and RsmZ. These small RNAs, predicted early to exist in the *Lp* genome (Kulkarni et al., 2006), are now experimentally confirmed to be transcribed and to bind to CsrA, un-blocking the translation of transcripts encoding transmissive traits (Rasis and Segal, 2009; Sahr et al., 2009). Optimal expression of *rsmY/Z* requires RpoS, confirming the previously suggested link between LetA/S, CsrA, and RpoS within the pathway that regulates differentiation in *Lp* (Hovel-Miner et al., 2009; Rasis and Segal, 2009).

A picture has recently emerged, in which CsrA seems to play an important role in regulating the expression and (or) activity of type IV secretion systems (T4SSs) in *Lp*, including the Dot/Icm virulence system (Rasis and Segal, 2009; Sahr et al., 2009; Nevo et al., 2014). Therefore, the correlation between differentiation and expression of virulence continues to be strengthened. A puzzling fact about the *Lp* pangenome is that it encodes, in a strain-dependent manner, several T4SSs whose genes are either stably integrated into the chromosome, or found within integrative and conjugative elements (ICE). For instance, strain 130b (also known as AA100), carry as many as six T4SSs (Schroeder et al., 2010), five of which reside in horizontally acquired, mobile genetic elements (Gómez-Valero et al., 2011; Wee et al., 2013). All these horizontally acquired T4SSs carry with them homologs of CsrA (Brassinga et al., 2003; Gómez-Valero et al., 2011; Wee et al., 2013; Flynn and Swanson, 2014). Therefore, the number of *csrA* copies would vary between strains, depending on how many genomic island-encoded T4SSs are present. Assuming that there might be functional differences between the various CsrAs present in a given strain, their regulatory flexibility could be astounding. However, it remains to be determined whether this is actually the case or not.

#### FliA and FleQ

Motility and differentiation are closely linked, simply because the differentiated transmissive *Lp* forms (SPFs and MIFs) are motile, whereas the replicative *Lp* forms (EPFs and RPFs) are not. It has been known that the flagellar sigma factor FliA is required both for the synthesis of flagella and for actual motility, as well as for achieving full virulence (Hammer et al., 2002; Heuner et al., 2002). It is thus not surprising that FliA, and its regulator FleQ (Albert-Weissenberger et al., 2010; Schulz et al., 2012), have found a place within the regulatory pathway of *Lp* differentiation, located downstream of CsrA (Heuner and Albert-Weissenberger, 2008; Sahr et al., 2009). Recent reports indicate that both ppGpp and DksA are required for the activation of the *fliA* promoter (Dalebroux et al., 2010), and have confirmed the role of FliA in virulence, showing that *fliA* mutants cannot compete with wild-type *Lp* during a co-culture assay in *A. castellanii* (Schulz et al., 2012).

#### DsbA

The bifunctional periplasmic disulfide bond oxidoreductase/isomerase of *Lp*, DsbA, modifies proteins by catalyzing the formation of disulfide bonds between cysteines (Kpadeh et al., 2013). In *Lp* there are two DsbA proteins, the non-essential DsbA1 and the essential and bifunctional DsbA2. When *dsbA2* is modified at the region encoding its active redox site, losses in *Lp* infectivity, intracellular growth and motility are observed (Jameson-Lee et al., 2011). Expression of native *dsbA2* was necessary for virulence and motility, as *Lp* carrying the defective *dsbA2* did not express flagellin and was deficient in Dot/Icm-dependent haemolysis (Jameson-Lee et al., 2011). It thus seems reasonable to propose that DsbA2 plays a role in *Lp* differentiation, as motility and the expression of a functional Dot/Icm system are hallmarks of transmissive *Lp* forms (SPFs and MIFs).

#### Integration host factor (IHF)

IHF is a heterodimeric DNA-binding protein that by virtue of its DNA-bending ability regulates transcription and recombination in bacteria (reviewed by Dorman, 2009). In *Lp*, IHF participates in the RPF-to-MIF differentiation (Morash et al., 2009) by an unknown mechanism. It is known that RpoS positively regulates the expression of the *ihfA* and *ihfB* genes, and that IHF acts as a positive autoregulator of expression (Pitre et al., 2013). In fact, experimentally confirmed binding sites for RpoS and IHF have been identified in the promoter regions of *ihfA* and *ihfB*. Puzzlingly, the DNA binding sites for the LetA response regulator (see above) and IHF have similar consensus sequences, suggesting that LetA and IHF do compete for these sites. Support for this notion comes from the fact that LetA negatively regulates the transcription of *ihfA* and *ihfB* (Pitre et al., 2013). Finally, IHF was found to cooperate with LetA in the induction of transcription of RsmY and RsmZ, further implicating IHF as a regulator of *Lp* differentiation. IHF is the third regulator of *Lp* differentiation (the other two being RpoS and LetA) for which mutants do not fully differentiate into MIFs (by morphological criteria), and grow in mammalian cells but not in amoeba (Morash et al., 2009). Thus, we would like to reiterate here our view that *Lp* is under strong selective pressure to differentiate into MIFs inside protozoa, but not in mammalian cells (Faulkner et al., 2008).

## Dissecting Lp's developmental network

### The various Lp developmental cycles and forms

In this section we have dissected *Lp*'s developmental network into five separate cycles, to highlight the main characteristics and biological relevance of key developmental forms found in the freshwater environment. As explained above (Section The Developmental Network of *Lp* and Why It is Necessary to Dissect It), trying to represent *Lp*'s developmental network in a single diagram would be overchallenging. Therefore, we have grouped closely related forms (Table 1) (on the basis of their direct biological relationships and presence in the same environmental niche) that sequentially differentiate into each other, to define each cycle. We believe that the cycles presented cover all the known aspects of *Lp*'s developmental biology. Although in our previous review (Garduño et al., 2008) we discussed FFs as a possible variation of SPFs, FFs have proven to be significantly different from bacillary *Lp* forms, particularly in the way they interact with host cells. In addition, filamentation in *Lp* often occurs in a growth phase-independent manner, thereby warranting the developmental separation of FFs and SPFs, as presented here.

#### EPFs and SPFs—the Lp extracellular growth cycle

**Figure 3:**
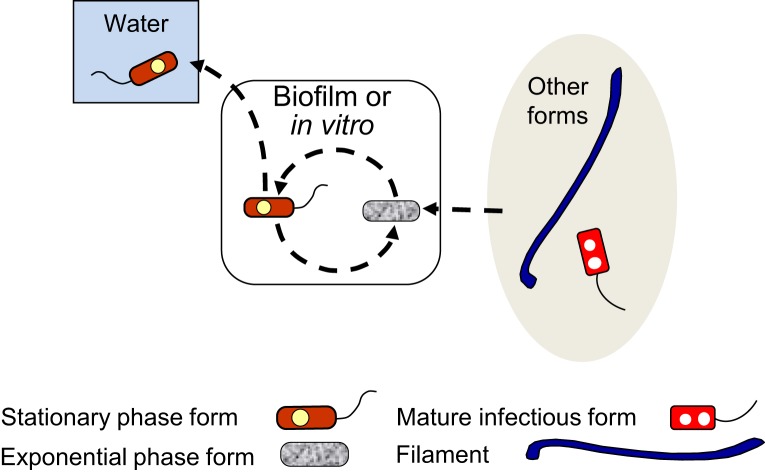
**Schematic representation of the exponential phase form (EPF)–stationary phase form (SPF) biphasic developmental cycle (dashed line arrows)**. EPFs and SPFs simply alternate into each other when *L. pneumophila* grows extracellularly, but SPFs can initiate intracellular cycles (**Figure 5**), or be internalized by ciliates of the genus *Tetrahymena* inside which they differentiate into mature infectious forms (**Figure 7**). SPFs produced in natural biofilms are likely to enter the water environment as planktonic free forms. SPFs and EPFs can produce filaments (**Figure 4**) and viable but non-culturable cells (**Figure 6**). Filaments and mature infectious forms are known to be able to differentiate into EPFs *in vitro*, entering the biphasic developmental cycle.

The biphasic extracellular growth cycle that alternates between EPFs and SPFs is schematically represented in Figure 3. This cycle happens in artificial, nutrient-rich culture media, and allegedly, within natural microbial communities, e.g., biofilms, where *Lp* could grow at the expense of dead microorganisms (Temmerman et al., 2006) or utilize nutrients released by other bacteria and (or) photosynthetic organisms (Tison et al., 1980; Pope et al., 1982; Bohach and Snyder, 1983a; Wadowsky and Yee, 1983, 1985; Hume and Hann, 1984a; Stout et al., 1985, 1986; Tison, 1987), onto which *Lp* might even physically attach (Bohach and Snyder, 1983b; Hume and Hann, 1984b).

A substantial body of experimental data has been obtained for the differentiation of EPFs and SPFs produced *in vitro* (in broth or agar cultures), but to the best of our knowledge, no experimentation has been reported on the differentiation of naturally produced EPFs and SPFs. *In vitro* EPFs have a typical Gram-negative envelope ultrastructure (Chandler et al., 1979; Faulkner and Garduño, 2002), and appear as slender short rods with a rather homogeneous cell size. The EPF is the *Lp* form that actively replicates in nutrient-rich media at a rate that varies according to growth conditions. EPFs utilize amino acids as their primary carbon and energy source, and are auxotrophic for cysteine (Ewann and Hoffman, 2006; Hoffman, 2008). Therefore, EPFs must rely on gluconeogenesis to synthesize the sugar precursors required for cell wall synthesis (Hoffman, 2008). Although synthetic media have been formulated for *Lp*, e.g., chemically defined media (Pine et al., 1986, and references within), the best growth of EPFs is always obtained in complex media with added yeast extract. The EPF is reportedly unable to initiate infections in macrophages and does not effectively avoid fusion with lysosomes (Joshi et al., 2001). Furthermore, the EPF is tolerant to salt, a phenotype that has been historically associated with avirulence in *Lp*. Consequently, the EPF is considered to be the replicative, non-infectious *Lp* form produced extracellularly.

In contrast, the SPF is infectious, morphologically heterogeneous (Chandler et al., 1979), and shows partial morphological differentiation features (Garduño et al., 2002) including the presence of cytoplasmic inclusions and invaginations of the inner membrane into the cytoplasm (Faulkner and Garduño, 2002). SPFs express transmissive traits and effectively initiate infections in macrophages, departing from the endocytic pathway shortly after internalization to establish a replicative vacuole (Joshi et al., 2001). The net gain in *Lp* cell numbers in the stationary growth phase is null or negative, however, it is virtually impossible to determine whether a proportion of SPFs in a *Lp* culture actually replicate or not. In spite of these technicalities, the SPF is generally considered non-replicative. As indicated in Section Background, the SPF has constituted the model *Lp* form for studying the molecular mechanisms of *Lp* differentiation into transmissive forms. We have determined that SPFs are metabolically active, consume oxygen in the presence of organic substrates, are infectious to a variety of mammalian cells in culture (Garduño et al., 2002), and remain culturable for long periods in water at room temperature (Al-Bana et al., 2014).

Although SPFs are confirmed transmissive forms of *Lp*, they show many differences with the transmissive *Lp* form produced intracellularly, i.e., MIFs. These differences have been repeatedly emphasized (Garduño et al., 2002, 2008; Faulkner and Garduño, 2002; Garduño, 2008) and will not be reiterated here. However, it is worth mentioning here that SPFs directly differentiate into MIFs inside food vacuoles of the ciliate *Tetrahymena* (Faulkner et al., 2008, also see Section The Cycle of Packaged *Lp* Forms below), indicating that these two forms are developmentally linked; the SPF being a stable differentiation intermediate between EPFs and MIFs.

In nature, EPFs and SPFs would be likely produced within biofilms, from which they would be released into the freshwater environment. However, these naturally produced forms could have different characteristics in relation to EPFs and SPFs produced *in vitro*. As transmissive *Lp* extracellular forms, SPFs have the potential for causing disease in humans. Also, naturally produced SPFs could initiate infections in freshwater amoeba and be ingested by ciliates, within which they would replicate and (or) differentiate to be released into the water environment as free or pelleted MIFs (Berk et al., 1998; Faulkner et al., 2008).

#### Filamentous forms (FFs)

**Figure 4:**
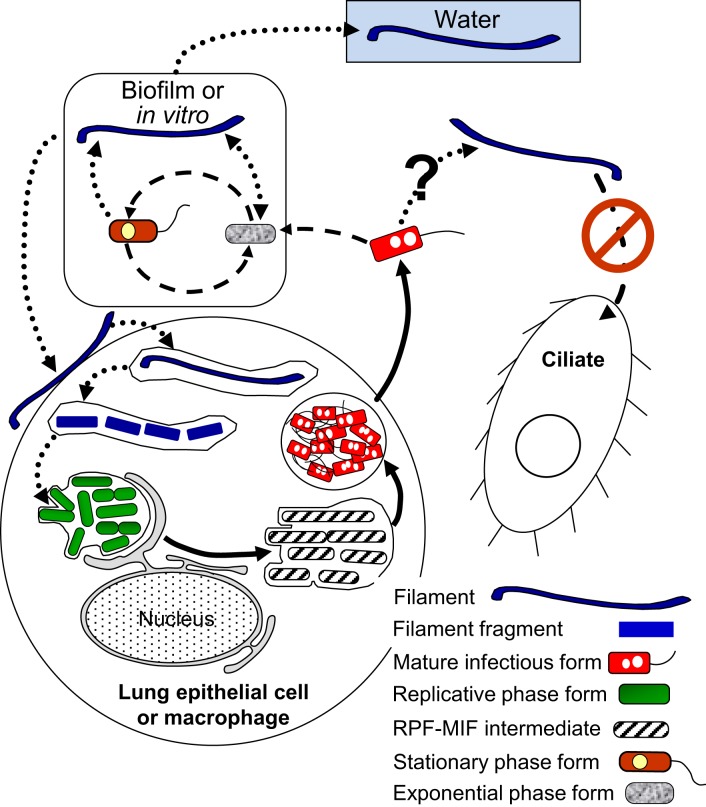
**Schematic representation of the multiphasic developmental cycle of filamentous forms (FFs) (round-dotted line arrows)**. Bacillary exponential phase forms and stationary phase forms produce FFs *in vitro* mainly in response to stress, and filamentation enhances biofilm-formation. FFs are infectious to lung epithelial cells and macrophages. As a consequence of the internalization of FFs by host cells FFs fragment to produce bacillary forms and eventually RPFs. FFs also differentiate into exponential phase forms *in vitro*. It is not known whether mature infectious forms can produce FFs, but free FFs are not internalized by *Tetrahymena* ciliates. Dashed line arrows, solid line arrows, and dash-dot patterned arrows are used to depict steps of the SPF–EPF developmental cycle (Figure 3), the MIF–RPF intracellular developmental cycle (Figure 5) and the ciliate-pellets developmental cycle (**Figure 7**), respectively.

We have summarized the developmental links of FFs in Figure 4. An anecdotal curiosity is that the very first picture of *Lp* ever published, prominently portrays a FF (McDade et al., 1977). However, the mechanisms that control *Lp* filamentation are poorly understood, as are its potential biological benefits.

Filamentation correlates with enhanced infectivity, persistence and pathogenesis in uropathogenic *E. coli* (UPEC) and *Proteus mirabilis* (Allison et al., 1994; Rosen et al., 2007). The gene *sulA* (encoding a cell cycle check-point protein that is part of the SOS response to stress) mediates filamentation of UPEC *in vivo*, and subvert innate immunity, confirming that a correlation exists between filamentation and pathogenesis (Justice et al., 2006). While *Lp* lacks the *sulA* gene and a *bona fide* SOS response (Charpentier et al., 2011). *Lp*'s differentiation into FFs is still linked to stressful signals such as nutrient limitation (Warren and Miller, 1979), the presence of antibiotics (Smalley et al., 1980; Elliott and Rodgers, 1985), high temperature (Piao et al., 2006), or exposure to UV radiation (Charpentier et al., 2011).

One clue that supports the developmental nature of filamentation comes from a study showing that overexpression of CsrA (the RNA-binding inhibitor of transcript translation, and a master regulator of *Lp* differentiation, Figure 2) enhances the production of *Lp* filaments in the post-exponential growth phase *in vitro* (Fettes et al., 2001). However, no mechanistic details on how CsrA regulates cell elongation are available. Other gene products implicated in the production of FFs are the HtpB chaperonin and the putative spermidine transporter PotD. Overexpression of HtpB leads to filamentation in *Lp* and *E. coli* (Garduño and Chong, 2013), and deletion of *potD* completely inhibits filamentation in stationary phase, while the *pot* operon promoter is highly activated in FFs (Nasrallah et al., 2014). Nonetheless, as for CsrA, no mechanism on how HtpB and PotD induce filamentation has been elucidated.

FFs have been observed in the water environment, in lung tissue and in clinical bronchial lavages (Rodgers et al., 1978; Prashar et al., 2012), and we now know that they can initiate intracellular infections in lung epithelial cells (Prashar et al., 2012) and macrophages (Prashar et al., 2013). In fact, the survival of filaments in macrophages correlates with length, so that the longest filaments are the most prone to replicate intracellularly (Prashar et al., 2013). In lung epithelial cells and macrophages, the uptake and early intracellular trafficking mechanisms of FFs are different from those established for bacillary *Lp* forms, and involve β 1-integrin and E-cadherin as well as unique membrane, actin and vesicular trafficking rearrangements (Prashar et al., 2012, 2013). These mechanistic differences between bacillary forms and FFs, suggest that FFs express unique bacterial cell surface molecules not present in bacillary *Lp* forms (none of which have been as yet identified), and (or) that the number and presentation of surface proteins is unique due to the dramatically increased surface of FFs.

A larger surface area would also favor the secretion and presentation of extracellular matrix materials required for the formation of biofilms. It is thus not surprising that long FFs actually produce robust *Lp* micelial mat-like biofilms in a temperature- and surface-type-dependent manner (Piao et al., 2006). FFs are not taken up by the ciliate *Tetrahymena tropicalis*, suggesting that by differentiating into FFs, *Lp* could avoid predation by bacteriovorous protozoa that do not support its intracellular growth (Berk et al., 2008). However, the effect of filamentation on the ability of protists to engulf *Lp* has not been studied in *Lp*'s natural environment. Although preliminary observations in our lab indicate the ability of *A. castellanii* to ingest FFs, the interaction of FFs with freshwater amoebae remains understudied and deserves further attention.

A fascinating event is the recently observed intracellular fragmentation of filaments (i.e., the differentiation of FFs into RPFs), which occurs as a consequence of FF uptake by macrophages (Prashar et al., 2013). This process also occurs extracellularly *in vitro* (Piao et al., 2006) where FFs differentiate into EPFs. Filaments are reportedly produced from EPFs and SPFs, but production of FFs by other *Lp* forms has not been reported. Finally, we would like to speculate on the significance of FFs in the transmission of Legionnaires' disease. Perhaps in the late stages of the disease, after *Lp* numbers in the lung have been amplified through replication in alveolar macrophages and patients develop a fever (inducing HtpB expression through high temperature), FFs could be commonly present and preferentially be taken by lung epithelial cells, which in turn could serve as reservoirs from which *Lp* could re-enter the environment.

#### RPFs and MIFs—the Lp intracellular growth cycles

**Figure 5:**
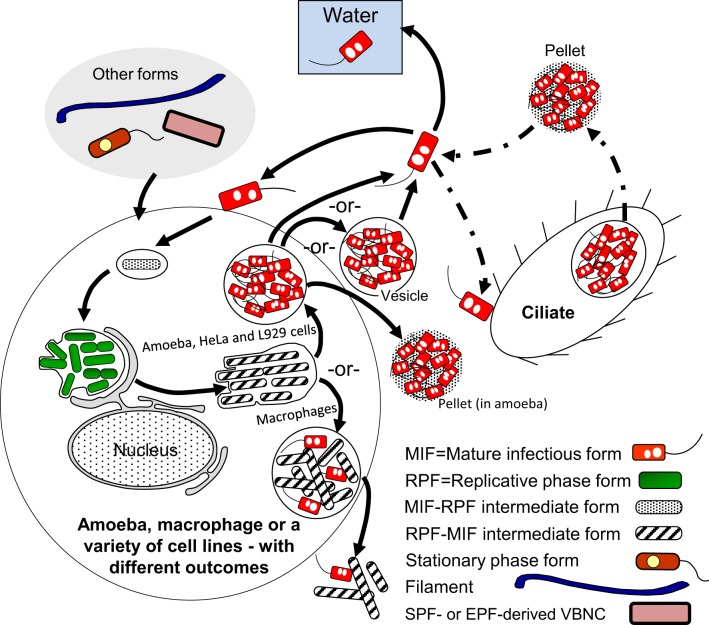
**Schematic representation of the replicative phase form (RPF)–mature infectious form (MIF) multiphasic developmental cycle (solid line arrows), which involves distinct morphological intermediate forms between MIFs and RPFs, and between RPFs and MIFs**. The cycle branch that happens in the ciliate *Tetrahymena* results in pellets of MIFs, but does not involve bacterial replication. The link to other cycles occurs when extracellular forms (“other forms” oval) initiate an intracellular infection that results in differentiation into RPFs, replication, and later differentiation into MIFs. MIFs, but not RPFs, persist in the water environment. It seems reasonable to surmise that if intracellular growth is the primary means of *L. pneumophila* replication in nature, MIFs would be the most abundant *Lp* transmissive form in the water environment. The dash-dot patterned arrows are used to depict steps of the ciliate-pellets developmental cycle (**Figure 7**).

A general representation of the intracellular developmental cycle depicted in Figure 5 is made possible by the remarkable conservation of intracellular events that characterize *Lp* infections, as described in human monocytes (Horwitz, 1983), mouse macrophages (Yamamoto et al., 1992), several mammalian cell lines (Oldham and Rodgers, 1985) including HeLa cells (Garduño et al., 1998), as well as different species of amoeba (Fields et al., 1989; Abu Kwaik, 1996; Solomon et al., 2000; Greub and Raoult, 2003; Lu and Clarke, 2005). Central in this general intracellular cycle is the MIF, initially named the mature intracellular form (Garduño et al., 1998) and subsequently renamed mature infectious form, to reflect the fact that MIFs persist in the extracellular environment as the predominant transmissive form of *Lp*. Thus, by our definition, the *Lp* progeny produced as a result of an intracellular growth cycle (see Section Extracellular vs. Intracellular *Lp*'s Developmental Cycles above) would be MIFs, which depending on the type of host cell infected, could have reached different developmental endpoints and exit as either free MIFs (which seems to be the most common mechanism, as described by Rowbotham, 1986), MIFs in host-derived membrane-bound vesicles [as observed in HeLa cells (Garduño et al., 1998) and amoeba (Rowbotham, 1986; Bouyer et al., 2007)], or pelleted MIFs (wrapped in a combination of multilamellar bodies produced by intra-endosomal budding in protozoa, and undigested bacterial membranous debris, Berk et al., 1998; Marchetti et al., 2004; Paquet et al., 2013).

MIFs exhibit several characteristic morphological and physiological features, which could be regarded as adaptations to both survive in the extracellular environment and remain infectious toward new potential hosts. These adaptations include resistance to environmental stressors (pH, detergents, chlorine, and antibiotics), a thickened cell wall that deviates from the characteristic Gram-negative ultrastructure, large and abundant cytoplasmic inclusions, metabolic dormancy, flagellation, and enhanced infectivity. Although MIFs were originally described in the context of the HeLa cell growth cycle, others (Greub and Raoult, 2003) and we (Abdelhady and Garduño, 2013, in addition to unpublished results) have confirmed that MIFs (with the same morphology and [or] general characteristics observed in HeLa cells) are also produced in amoeba. In addition, a retrospective look at publications regarding the intracellular growth of *Lp* in amoeba, starting with the careful observations of Rowbotham (1986), confirms beyond any doubt the natural production of MIFs in amoebal hosts (also reviewed by Garduño, 2008).

Once internalized by a new host, MIFs quickly adapt to the intracellular host environment and any possible host defense mechanisms triggered by their internalization. This adaptation is a pre-requisite for replication and primarily, albeit not exclusively, is mediated by the Dot/Icm type IV secretion system. The Icm/Dot system secretes a multitude of functionally redundant effectors that act at every stage of the infection process, beginning with the binding of *Lp* to cell surface receptors of the new host, and ending with the exit of progeny from the wasted host cell (reviewed by Hoffmann et al., 2014). In this respect, we have often argued that MIFs must be “infection-ready,” carrying a spring-loaded Dot/Icm system (set during the differentiation of RPFs into MIFs) that is released upon contact with a new host cell, allowing the establishment of an intracellular niche within minutes after internalization (Roy et al., 1998).

However, before fully exploiting the newly acquired intracellular niche and beginning replication, MIFs must first induce host mechanisms to transport (or themselves directly transport) nutrients from the host cell cytoplasm into the lumen of the replicative vacuole. Not until these nutrients (primarily amino acids) reach a threshold concentration inside the *Legionella*-containing vacuole (LCV), MIFs can differentiate into RPFs. Among these nutrients, *Lp* must be able to have access to iron (reviewed by Cianciotto, 2007), in addition to nucleosides (Fonseca et al., 2014), to initiate and maintain growth, but amino acids seem to be the primary triggers for differentiation into RPFs.

The collective experimental evidence that supports the role of amino acids in the MIF-to-RPF differentiation is as follows: (i) During *Lp* infection the host amino acid transporter SLC1A5 (putatively responsible for mobilizing cytoplasmic amino acids into the lumen of the LCV) is induced (Wieland et al., 2005). (ii) Human MM6 monocytes with a chemically inactivated, or post-transcriptionally silenced SLC1A5 transporter do not support the growth of *Lp* (Wieland et al., 2005). (iii) A transposon-insertion mutant with a defective amino acid transporter (PhtA, with high affinity for threonine) invades well but does not replicate in murine macrophages. This mutant is also defective at initiating growth *in vitro* (Sauer et al., 2005a). (iv) The growth defects of the *phtA Lp* mutant are reversed by supplying an excess of exogenous amino acids (particularly threonine). (v) The *phtA* mutant remains “locked” as a SPF, predominantly showing transmissive phenotypes (Sauer et al., 2005a). (vi) The concentration of free amino acids is increased in *Lp*-infected cells in an AnkB-dependent manner (Price et al., 2011); AnkB being a LetE-regulated effector of the Icm/Dot system (Figure 2) that promotes the degradation of ubiquitinated host cell proteins. (vii) *ankB* mutants cannot initiate intracellular replication in spite of being able to establish an apparently functional LCV, and persist in the LCV as a form that predominantly expresses transmissive phenotypes (Price et al., 2011). (viii) *ankB* mutants (but not a *dotA* mutant) can be rescued and initiate intracellular growth by the addition of exogenous amino acids (Price et al., 2011). (ix) The presence of arginine in the LCV induces major changes in *Lp* gene expression, mediated by the inhibition of ArgR (Figure 2), an important transcriptional regulator (Hovel-Miner et al., 2009).

RPFs have a morphology that is indistinguishable, at the ultrastructural level, from that documented for EPFs. That is, both RPFs and EPFs show an envelope ultrastructure that is typical of Gram-negative bacteria, an electron-dense cytoplasm rich in ribosomes, and a lack of cytoplasmic inclusions (Faulkner and Garduño, 2002). RPFs usually show an intimate interaction with the inner face of the LCV membrane. In transmission electron microscopy sections of *Lp*-infected cells, the LCV membrane closely follows the contour of the contained RPFs. This feature is displayed both in protozoan and in mammalian host cells, suggesting that the underlying mechanism involved is conserved among eukaryotes. We propose that such intimate interaction is related to the acquisition of nutrients by RPFs. In this respect, a supply of nutrients must be secured to sustain the active replication of RPFs.

Factors inferred to contribute to the flow of nutrients from the host cell into the LCV's lumen include the AnkB-mediated degradation of host proteins, which results in increased levels of available amino acids (recently reviewed by Price et al., 2014), the transport of amino acids across the LCV membrane (Wieland et al., 2005), and the characteristic association of the LCV with mitochondria and the endoplasmic reticulum (ER), first recognized in electron microscopy studies of *Lp*-infected human monocytes (Horwitz, 1983). We have demonstrated that the *Lp* chaperonin, HtpB, reaches the cytoplasm of host cells and associates with the LCV membrane (Nasrallah et al., 2011; Garduño and Chong, 2013). Furthermore, purified HtpB attached to polystyrene microbeads attracts mitochondria by an unknown mechanism (Chong et al., 2009). The secretion by *Lp* of an eukaryotic-like sphingosine-1-phosphate lyase, LegS2 (Degtyar et al., 2009) and a mitochondrial carrier protein, LncP (Dolezal et al., 2012), both of which localize to mitochondria after secretion, could induce nutrient leaching in the attracted mitochondria. In fact, the secreted LncP localizes to the inner membrane of host cell mitochondria, from where (by means of LncP's ability to transport nucleotides across proteoliposomes *in vitro*) it is speculated to help in the unidirectional flow of nutrients from mitochondria to the LCV lumen (Dolezal et al., 2012). If this would be the case, mitochondria could be the source of nucleotides to be taken up by RPFs from the LCV by the transporters PhtC/D (Fonseca et al., 2014).

A second experimentally demonstrated ability of HtpB that is related to nutrient acquisition is its specific interaction with host S-adenosyl methionine decarboxylase (SAMDC). We have proposed that this interaction contributes to the intracellular production of elevated levels of polyamines, in turn required for the optimal replication of RPFs (Nasrallah et al., 2011). Finally, the association of the LCV with the ER is thought to be a major contributor of nutrients for RPFs. However, in spite of intensive recent work showing the targeting of Dot/Icm effectors to the ER/Golgi/ER vesicular trafficking, and their specific effects on these processes (which are beyond the scope of this review), the flow of nutrients from the ER to the LCV has not been unequivocally demonstrated, except for the structural incorporation of ER-derived vesicles into the LCV and consequent derivation of LCV membrane from the ER, as reported by Tilney et al. (2001). That is, the cargo of ER-derived vesicles, and the ER membrane itself, could be sources of nutrients and lipids for RPFs.

Replication of RPFs usually result in LCVs that are “packed full” of *Lp* cells, which late in the infection cycle would differentiate into MIFs through a number of morphological intermediates, thereby closing the MIFs-RPFs intracellular growth cycle. One point to emphasize here is that according to Abdelhady and Garduño (2013) (see Section Extracellular vs. Intracellular *Lp*'s Developmental Cycles above), MIFs produced in different hosts could reach different developmental endpoints. This is not surprising because the *Lp* growth cycles followed in different host cells also show unique defining features. For instance, the late stage of *Lp*'s growth in murine macrophages is characterized by the fusion of lysosomes with, and the acidification of, the LCV (Sturgill-Koszycki and Swanson, 2000), whereas in human macrophages this does not happen (Wieland et al., 2004; Sauer et al., 2005b). Moreover, the intracellular environment must not be the same between different amoebal species, because these do not support growth of the same *Lp* serogroups (Rowbotham, 1980). An analysis of the transcriptome of *Lp* in a variety of host cells, in conjunction with a phenotypical characterization of the progeny produced (see Section Potential Molecular Markers for Detection of MIFs below), would significantly enhance our understanding of the impact that particular intracellular host environments have on the differentiation process of *Lp*.

Finally, differentiation of RPFs into MIFs must be a survival and (or) late growth requirement inside protozoa, but not inside mammalian cells. That is, *rpoS*. *letA*, and *ihfAB* mutants with defects in RPF-to-MIF differentiation (refer to Sections RpoS, Two-Component Regulatory Systems (LetA/S, PmrA/B, and LqsR/S), and Integration Host Factor (IHF) above) are able to grow well and release a partially differentiated progeny in mammalian cells, but not in amoeba (Hales and Shuman, 1999; Gal-Mor and Segal, 2003; Abu-Zant et al., 2006; Faulkner et al., 2008; Morash et al., 2009). These mutants are also completely digested in the food vacuoles of *Tetrahymena tropicalis* (Faulkner et al., 2008). Therefore, we propose that, in nature, the *Lp* forms most often found in the freshwater and moist soil environments must be fully differentiated MIFs produced as a result of *Lp*'s growth in protozoa. This would be in sharp contrast to what happens during replication in mammalian cells, where *Lp* is not under a strong selective pressure to differentiate, and would thus produce a mixture of MIFs with a variety of developmental maturities (Abdelhady and Garduño, 2013, Figure 5); a factor to consider in explaining (at least in part) the lack of person-to-person transmission of Legionnaires' disease.

#### Viable but not culturable cells (VBNCCs)

Being unable to sporulate, Gram-negative bacteria survive severe environmental stress by becoming dormant (reviewed by Oliver, 2010). This “standby mode” of survival, known as the viable but non-culturable (VBNC) state, is characterized by a physiological adjustment, perhaps similar to the stringent response, but with more profound consequences, i.e., loss of culturability. Key to the decision of including a discussion on VBNC *Lp* as a distinct developmental form, was to take a side on the controversy of whether the VBNC state results from differentiation or simply from cell injury (Nyström, 2003). That is, on the one hand, it has been argued that VBNC cells (VBNCCs) are no more than injured cells struggling to stay alive for as long as physiologically possible, thereby eliciting stress responses and repair mechanisms in a general manner. On the other hand, entry into the VBNC state is viewed as a purposeful physiological adaptation that requires a coordinated change in gene expression, regulated (at least in part) by the same factors that control stress responses and repair mechanisms. We subscribed to the latter, mainly because our own experimentation with VBNCCs derived from MIFs (that will be described further in this section), suggests that in the VBNC state *Lp* maintains a robust ultrastructure and physiology, which would be difficult to reconcile with the view of injured cells at the brink of death. Due to the important implications of VBNCCs in water quality control and detection of *Lp* in the context of public health, VBNC *Lp* has recently received increased attention. However, many gaps still exist in our understanding of *Lp* VBNCCs, in particular, the molecular mechanisms that orchestrate and control entry into, and exit from, the VBNC state.

**Figure 6:**
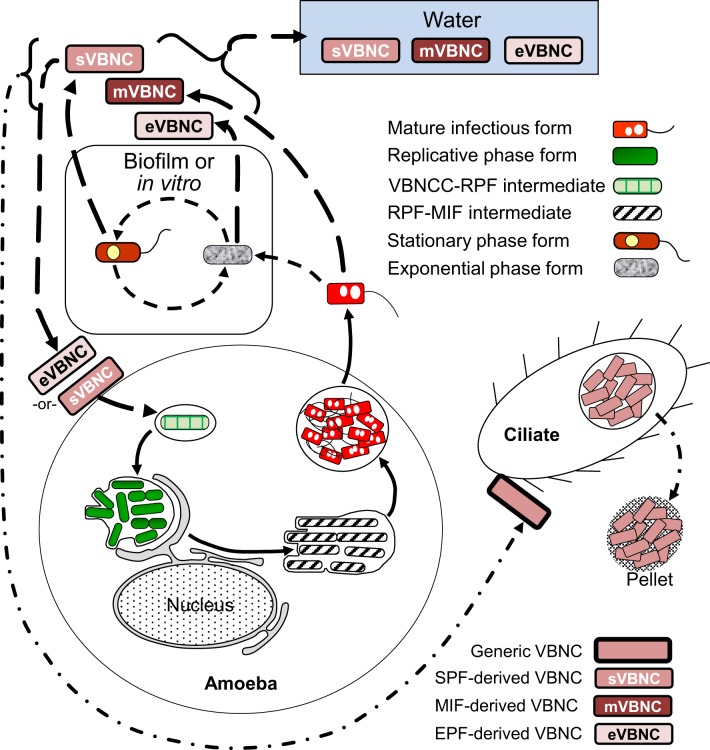
**Schematic representation of the developmental cycle of viable but non-culturable cells (VBNCCs) (long-dashed line arrows)**. Mature infectious forms (MIFs), stationary phase forms and exponential phase forms *in vitro* or in biofilms produce VBNCCs (mVBNC, sVBNC, and eVBNC, respectively) through either a natural process of attrition during cell senescence, or programmed differentiation. Internalization by amoebae resuscitates eVBNC and sVBNC cells, which then differentiate into replicative phase forms and produce a progeny of MIFs. Resuscitation of mVBNC cells has not been reported. mVBNC and sVBNC cells ingested by *Tetrahymena* ciliates survive in food vacuoles and produce pellets, but do not resuscitate in this host. Dashed line arrows, solid line arrows, and dash-dot patterned arrows are used to depict steps of the SPF–EPF developmental cycle (Figure 3), the MIF–RPF intracellular developmental cycle (Figure 5) and the ciliate-pellets developmental cycle (**Figure 7**), respectively.

The developmental links that we have identified for VBNCCs are shown in Figure 6. So far, we know that VBNCCs can be produced from EPFs (Ohno et al., 2003), SPFs (Ohno et al., 2003; Al-Bana et al., 2014) and MIFs (Al-Bana et al., 2014). The characteristics of these VBNCCs are defined by the developmental form that produces them and that is why we depict them as three different entities.

The triggers for VBNCC production (as previously noted for the production of FFs) are stress-related and numerous, but prolonged starvation in water is a natural condition (likely encountered by *Lp* on a regular basis) that consistently induces VBNCC formation in *Lp* (Steinert et al., 1997; Ohno et al., 2003; Al-Bana et al., 2014). Temperature increases and a reduction in the concentration of inorganic ions, results in significant shortening of the time required to enter the starvation-mediated VBNC state in water (Ohno et al., 2003; Al-Bana et al., 2014). Once formed, VBNCCs would persist in the water environment for extended periods, until they receive a signal to “wake up” from dormancy, a process known as VBNCC resuscitation.

In our work with VBNCCs derived from SPFs (SPF–VBNCCs) or MIFs (MIF–VBNCCs) in sterile tap water at 45°C (Al-Bana et al., 2014), we observed that cytoplasmic inclusions and a portion of the cytoplasmic material are consumed during the starvation period, so that VBNCCs become thin. Under the transmission electron microscope, the cytoplasm of SPF–VBNCCs shows numerous zones with low electron density and one or two electron-dense spots, and the outer membrane shows a wavy contour with small projections, suggesting that SPF–VBNCCs produce outer membrane vesicles. In contrast, MIF–VBNCCs maintain an electron-dense cytoplasm and an apparently intact envelope that shows the typical traits originally described for MIFs from HeLa cells. The proportion of starved MIFs that enter the VBNC state in water at 45°C is between 70–90%, with no significant drop in viability after 1 month. It is the combination of this high viability and ultrastructural preservation that made us conclude that formation of MIF–VBNCCs is a purposeful differentiation that *Lp* uses to cope with environmental stress. It only remains to demonstrate that MIF–VBNCCs can resuscitate with high efficiency into RPFs to close the MIF–VBNCC branch of the cycle. Thus, it would be interesting to test on MIF–VBNCCs the newly reported resuscitation method that incorporates the addition of organic scavengers of oxygen radicals (Ducret et al., 2014).

VBNCCs derived from SPFs or EPFs resuscitate in the presence of amoeba (Ohno et al., 2003; Al-Bana et al., 2014). Upon resuscitation in amoeba, it is not clear whether the differentiation of VBNCCs into RPFs includes the formation of intermediate FFs, but starved *Lp*, just before becoming unculturable, profusely produce filaments when placed on nutrient-rich BCYE plates and convert into EPFs (Al-Bana et al., 2014).

One final point in relation to VBNCCs is the controversial discussion of whether VBNC *Lp* is capable of causing Legionnaires' disease in humans. Although the potential for disease transmission exists, infection of mammalian cells by VBNCCs *in vitro* has not been experimentally demonstrated. Regardless, the potential resuscitation of VBNCCs by amoeba is sufficient to implicate VBNCCs as relevant to human health, as this would (i) allow for a repopulation of water systems by *Lp* MIFs following disinfection attempts, and (ii) inhalation of amoeba carrying VBNCCs, could potentially initiate an infection (Brieland et al., 1997).

#### The cycle of packaged Lp forms

**Figure 7:**
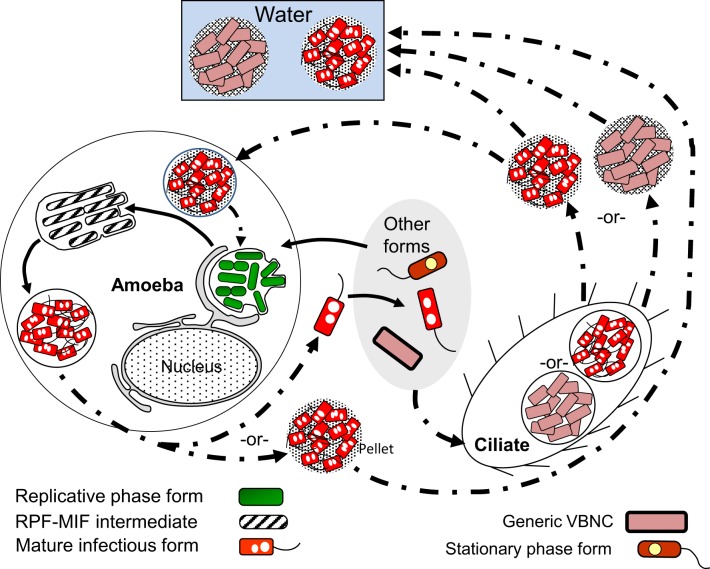
**Schematic representation of the ciliate-pellets cycle (dash-dot pattern line arrows) following packaging of *Lp* into pellets by ciliates and amoeba**. *Lp* cells inside pellets may be packaged in wraps of degraded *Lp* membranous material and (or) in some protozoa-produced matrix and multilamellar bodies, or in a combination of both. Pellets represent the contents of food vacuoles that have been emptied into the surrounding environment. The reader is reminded that stationary phase forms (SPFs) ingested by *Tetrahymena* ciliates rapidly differentiate into mature infectious forms inside food vacuoles, so that pellets of SPFs are not produced. Pellets should not be confused with free vesicles containing *Lp* progeny still inside a membrane-bound vacuole (e.g., Figure 5), released from lysed host cells (protozoan or mammalian). Presence of pellets in water might increase the infectious challenge encountered by vulnerable individuals. Solid line arrows represent steps of the RPF–MIF developmental cycle (Figure 5).

The last cycle to discuss here is the one in which ciliates of the genus *Tetrahymena*, as well as amoeba, participate by ingesting *Lp* cells into food vacuoles, and later expelling the content of such vacuoles in the form of “packaged” fecal pellets containing live *Lp* cells (Denoncourt et al., 2014). The pelleted live *Lp* cells would act as complex infectious particles that can initiate infections after being phagocytosed whole by either amoeba or macrophages, thus closing the cycle to commence a new one as RPFs (Figure 7). We decided to include this cycle among the developmental *Lp* network for two main reasons. First, the *Tetrahymena* food vacuoles promote the direct differentiation of SPFs into MIFs in the absence of intracellular replication (Faulkner et al., 2008). Second, packaged MIFs could be an effective way (in addition to biofilms) for *Lp* to persist in the water environment, or even in non-aqueous niches where resistance to desiccation would be afforded by the pellet configuration, and from where new infections could be initiated (in amoeba, or accidentally in humans) (Denoncourt et al., 2014). In this respect, packaged MIFs could be “the” infectious particle hypothesized by Rowbotham (1980, 1986) to transmit Legionnaires' disease, spreading *Lp* from the environment to the human lung in one large installment.

VBNCCs are also pelleted by *Tetrahymena tropicalis* and *Tetrahymena thermophila* (Al-Bana et al., 2014). The pelleted VBNCCs were shown to (i) remain viable by means of a vital fluorescent stain, (ii) preserve their envelope integrity, (iii) become Gimenez-positive (i.e., acquire a bright red color after the Gimenez stain) and (iv) not become structurally degraded (i.e., digested by the ciliates). Resuscitation in *Acanthamoeba* was possible for pelleted SPF–VBNCCs, but not for MIF–VBNCCs, indicating that pelleted SPF–VBNCCs remain infectious.

## Practical implications

### Searching for differentiation markers applicable to the detection of *Lp* forms

When we pay attention to what is in all the “Water” boxes of the five developmental cycles presented above (Figures 3–7) it becomes apparent that not all the legionellae found in the water environment are the same. Yet, when *Lp* is detected in water samples, it is generally assumed that it exists in a single form; an exception being the awareness that has existed about VBNCCs (Hussong et al., 1987; Hwang et al., 2006; Slimani et al., 2012). But even if one would recognize and accept the existence of the many *Lp* forms, the question is how could these forms be detected and distinguished from each other? Currently available methods are not geared to distinguish between *Lp* forms, except—again—for the practical efforts made to distinguish and quantify VBNCCs using flow (Keserue et al., 2012) or solid-phase (Parthuisot et al., 2011) cytometry. Thus, when *Lp* is detected and quantified in water samples, one cannot tell which *Lp* forms are present, nor their proportions. Because the ecology of each of these forms (as suggested by their developmental cycles) seems to be different (as is their potential to transmit disease), it would be highly desirable to have some useful tools in our analytical toolbox, which could allow us to detect the many *Lp* forms present in water. What follows in the last part of this review, is an account of possible markers (morphological and molecular) that could be used to identify some of the *Lp* forms. It should be noted that *Lp* forms have to be detected in the absence of any culturing step, not to change the developmental stage of the forms present.

#### Potential molecular markers for detection of MIFs

The MIF-associated protein MagA was first discovered as a 24-kDa protein induced during macrophage infection, which was annotated as “Mip-like protein” (Miyamoto et al., 1993). We re-encountered the protein as one consistently induced to high levels in *Lp* cells placed in water, and later found that its expression was linked to the differentiation of *Lp* into transmissive forms (SPFs and MIFs) (Hiltz et al., 2004), with very high levels produced in MIFs (Garduño et al., 2002; Hiltz et al., 2004). We renamed the protein MagA, to avoid confusion with the macrophage infection potentiatior protein Mip (Bachman and Swanson, 2004; Hiltz et al., 2004). However, it turns out that MagA is not a good marker for MIF identification, mainly because its encoding gene is exclusively carried in the Philadelphia *Lp* lineage, as part of a genomic island (Brassinga et al., 2003) recently identified as a mobile integrative conjugative element that confers fitness advantages (Flynn and Swanson, 2014). In addition, MagA is a cytoplasmic protein that would be difficult to target with antibodies for fast detection without culture.

When comparing the 2-D protein profiles of SPFs and MIFs (derived from the Philadelphia-1 strain SVir), we identified 17 MIF-specific protein spots (Garduño et al., 2002). MagA was not picked in this comparison because it is also expressed by SPFs (albeit at much lower levels). When identified by mass spectrometry, some of the spots showed identity to predicted hypothetical proteins (encoded by *lpg0563, 2526, 2755*), and some others included the small heat shock protein HspC2, Mip, 50S ribosomal protein L9, and the 27-kDa outer membrane protein, as well as a lipase, glycyl-tRNA synthetase, and glutaryl-CoA dehydrogenase. However, no further work has been completed to confirm whether these proteins are indeed exclusively expressed in MIFs.

Additional potential markers useful in detecting MIFs could emerge from transcriptomic data obtained 30–60 min after SPFs have been ingested by *Tetrahymena tropicalis*, since this is the time at which SPFs are undergoing a direct differentiation into MIFs, inside the ciliate's food vacuoles (Faulkner et al., 2008). In collaboration with C. Buchrieser (Institut Pasteur, Paris) we have completed this work, but the complete microarray data obtained will be published elsewhere. It was interesting, nonetheless, to find that during the transition into MIFs inside the ciliates, SPFs induced the expression of genes encoding enzymes involved in carbohydrate metabolism. One of these genes (*lpg1669*), encoding a putative amylase, was also found to be grossly transcribed (>150-fold) in amoeba when the microarray data was confirmed using quantitative, reverse-transcriptase PCR. Of great interest is the observation that Lpg1669 was not induced in macrophages, suggesting that in searching for MIF-specific differentiation markers, it might be productive (besides looking at differences between SP and MIF) to look at differences between MIFs produced in different hosts. By comparing the published microarray data for the transcriptome of *Lp* grown in amoeba (Brüggemann et al., 2006) against that of *Lp* grown in macrophages (Faucher et al., 2011) we have identified a list of differentially expressed genes. It should be noted that the transcriptome studies mentioned above use different time points as reference (0 vs. 8 h post-infection as the undifferentiated control). In spite of this limitation, we believe that the analysis is valuable, mainly because the data still highlight major gene expression differences in MIFs obtained in different hosts. Therefore, we focused on a short list of genes that are: (i) induced more than 2-fold in amoeba, and (ii) either repressed or unchanged in macrophages (Table 2). Reasoning that the MIFs present in the water environment would have emerged from protozoa and not from macrophages, some of the highly induced genes showed in Table 2 could be useful markers for MIF detection in water samples by reverse transcription PCR, or by immunoaffinity reagents to their gene products.

**Table 2:** **Short list of *Lp* genes whose transcription is selectively upregulated in amoebae (MIF transcript level/RPF transcript level >2), but down-regulated or unchanged in human macrophages (MIF transcript level/SPF transcript level <2)**.

**Gene ID**	**Description**	***A. castellanii*^a^**	**Human macrophages^b^**
		**T_14_/T_8_**	**T_18_/T_0_**
*lpg0910*	Enhanced entry protein A	20.25	0.99
*lpg0818*	ATP-dependent Clp A protease	4.66	0.81
*lpg0891*	Sensory box protein/GGDEF/EAL domains	10.93	0.83
*lpg1356*	Enhanced entry protein C	11.63	0.77
*lpg1491*	Lem9 (Dot/Icm effector)	15.78	1.78
*lpg0670*	Hypothetical protein	8.94	0.66
*lpg1669*	Putative α-amylase	17.88	0.87
*lpg2228*	3-oxoacyl ACP synthase III	7.62	1.55
*lpg2316*	3-hydroxybutyrate dehydrogenase	8.82	0.74
*lpg1540*	Universal stress protein A	4.66	1.94
*lpg2348*	Superoxide dismutase SodC	6.96	0.84
*lpg2955*	Integration host factor HipB	8.94	0.79
*lpg2971*	Malate dehydrogenase	12.13	0.60
*lpg1639*	Hypothetical protein	13.74	1.56
*lpg0279*	Hypothetical protein	9.45	0.67
*lpg2495*	Homospermidine synthase	7.26	1.06
*lpg1887*	Hypothetical protein	11.00	0.91

*^a^Data were obtained from Brüggemann et al. (2006), where T_14_ = 14 h after infection, post-replicative phase (MIFs), and T_8_ = 8 h after infection, replicative phase (RPFs)*.

*^b^Data were obtained from Faucher et al. (2011), where T_18_ = 18 h after infection, post-replicative phase (MIFs), and T_0_ = infection at zero time with SPFs grown in vitro*.

#### Detection of VBNCCs

As mentioned in Section Searching for Differentiation Markers Applicable to the Detection of *Lp* Forms above, there are a number of published methods that have potential applications in the detection of *Lp* VBNCCs. These methods exploit the fact that VBNCCs must be positively stained with vital stains while not be able to grow on BCYE agar. However, recent interest has been raised in examining the proteome of VBNCCs (Alleron et al., 2013, Antje Flieger, Robert Koch Institute—personal communication; R. Garduño—unpublished results). Using 2-D gels of ^35^S-labeled proteins, Alleron et al. (2013) identified nine spots that were present in VBNCCs, but not in SPFs, which included some potential virulence-related proteins. Interestingly, among these nine VBNCC proteins, Mip, the 27-kDa outer membrane protein, and the 50S ribosomal protein L9, were three proteins also found in MIFs as part of our proteomic study (see Section Potential Molecular Markers for Detection of MIFs above), indicating that these particular proteins are not VBNCC-specific. Confirmation of whether the other identified proteins are VBNCC-specific would be useful in potentially improving VBNCC detection, by incorporating the labeling of VBNCC-specific proteins in current cytometry-based methods (Parthuisot et al., 2011; Keserue et al., 2012).

#### Morphological markers

The most obvious application of morphological markers would be in the detection of FFs and pellets of VBNCCs and MIFs, which would be easily distinguished by microscopy or cytometry. We have used an OmpS-specific antibody (Butler and Hoffman, 1990) and a secondary fluorescent antibody to immuno-label water samples concentrated by filtration through a 0.45 μm-pore membrane. This method easily renders *Lp* visible by fluorescence microscopy, under which FFs and *Lp* pellets are readily spotted.

## Conclusion

*Lp* differentiates into 14 developmental forms reported to date (and likely new ones will be described) following a complex developmental network that has been defined and described in this review. Therefore, we urge readers to abandon the common depiction of *Lp*'s differentiation as a biphasic developmental process that alternates between replicative and transmissive forms, mainly because this view is an oversimplification of the actual process.

It is our prediction that in the near future, novel developments will make possible the detection of key *Lp* forms found in water. New knowledge both on the proportion in which these forms appear in different water environments (e.g., cooling towers vs. potable water systems), and on their relative infectivity to cells in culture (or ideally, infectivity to animal models via aerosolization) could help immensely in the proper assessment of risk and the effective control of Legionnaires' disease outbreaks.

### Conflict of interest statement

The authors declare that the research was conducted in the absence of any commercial or financial relationships that could be construed as a potential conflict of interest.
